# Editorial: Research implications on microbial virulence factors, resistance, and new therapeutic strategies in the context of future infectious disease therapies

**DOI:** 10.3389/fcimb.2024.1406119

**Published:** 2024-06-07

**Authors:** Mariusz Dyląg, Brankica Filipić, Daria Augustyniak, Marina T. Milenković

**Affiliations:** ^1^ Department of Mycology and Genetics, Faculty of Biological Sciences, University of Wroclaw, Wroclaw, Poland; ^2^ Department of Microbiology and Immunology, Faculty of Pharmacy, University of Belgrade, Belgrade, Serbia; ^3^ Department of Pathogen Biology and Immunology, Faculty of Biological Sciences, University of Wroclaw, Wroclaw, Serbia

**Keywords:** virulence factors, resistance, novel therapeutic strategies, infectious diseases, epidemiology, culture-independent diagnostics

Fatal infections are related to virulence factors and resistance which are crucial for pathogenesis (Boneca, 2021; de Kraker and Lipsitch, 2022; Gupta, 2024). The main goal of our Research Topic is to gather the latest articles that address microbial virulence factors, resistance, and effective solutions to overcome such phenotypes. Additionally, we aim to revive the discussion on the changing epidemiology and emerging therapeutic strategies for infectious diseases. In this Research Topic, we will discuss recent developments and innovative approaches in this field, and we believe that potential readers will discover the latest findings in line with the proposed theme. In the context of the changing epidemiology, Bakuła et al. conducted a study of the tuberculosis (TB) situation in Poland and northeastern Europe. They assessed the susceptibility and resistance of the tested TB isolates to the available anti-tuberculosis drugs. Their analyses focused on the genotypic diversity of multidrug-resistant (MDR) and drug-susceptible (DS) *Mycobacterium tuberculosis* strains isolated in this region of Europe. The results obtained from the susceptibility profiles are highly relevant in an epidemiological context. The authors showed that between 2018 and 2021, the Beijing and Haarlem genotype families were the most common isolates among MDR-TB strains. In Poland the Beijing genotype family was the most prevalent (61.5%) and its prevalence is increasing due to imports from countries of the former Soviet Union (Bakuła et al.). In addition to various *in vitro* and *in silico* studies on *M. tuberculosis*, Wei et al. have made significant findings related to cognition. These authors performed a transcriptional analysis of human peripheral blood mononuclear cells (PBMC) stimulated with *M. tuberculosis* heat-resistant antigen (Mtb-Hag). This antigen is known to stimulate γδT cells to trigger an immune response against tuberculosis. The authors analyzed the PBMC samples using high-throughput RNA sequencing, and compared the results from Mtb-Hag-stimulated and control samples. They also looked at available information in proteomic and metabolomic gene ontology (GO), Kyoto Encyclopedia of Genomes (KEGG), and PPI protein interaction network databases. Their analysis revealed 597 differentially expressed genes in the PBMC samples, which were mainly associated with TNF, IL-17, JAK-STAT, NF-κB signaling pathways, and cytokine-cytokine receptor interactions. The results obtained from the analysis of transcriptomes of PBMC stimulated by Mtb-Hag, may be a prelude to exploring the intracellular immune mechanisms against *M. tuberculosis* and improving the vaccine against tuberculosis (5). The studies by Huang et al. and Li et al. also fit very well into the theme of our Research Topic. The article by Huang et al. addressed the identification of virulence traits and genes, the prediction of antibiotic resistance genes, and finally the phylogenetic relationship between *Kluyvera* and *Phytobacter*, species that are often misdiagnosed. Studies performed on *Phytobacter* in comparison with *Kluyvera* for the identification of specific virulence genes clbS, csgA-C, fliS, hsiB1_vipA and hsiC1_vipB allow to conclude that these are not present in the *Kluyvera* genome. Moreover, the authors proposed 11 core genes of *Kluyvera* that could serve as potential identification targets, and procedures based on average nucleotide identity (ANI) with *in silico* DNA-DNA hybridization (isDDH)dDDH for differentiation from species of the genus *Phytobacter*. This work is the first to address the evolution, pathogenicity, and drug resistance of two emerging pathogens. The authors highlighted the coexistence of ESBLs and carbapenem resistance genes, which were present in approximately 40% of the strains. This is a critical finding for understanding the phenomenon of drug resistance (Huang et al.). In turn, Li et al. described an important association between the type VI secretion system (T6SS) and drug resistance in *Acinetobacter baumannii*. They discovered three T6SS core genes, namely tssB, tssD (hcp), and tssM, which are involved in the drug resistance and virulence of *A. baumannii*. These researchers evaluated the role of three T6SS core components, TssB, TssD (Hcp), and TssM, in terms of biofilm formation, bacterial competition, normal human serum resistance, and host colonization. All of the presented results may provide potential therapeutic and vaccine targets for the control of *A. baumannii* infections (Li et al.). In the context of the search for new therapeutic strategies focusing on antimicrobials targeting virulence factors, the article by Yin et al. is very relevant. These authors discovered that epigallocatechin-3-gallate (EGCG) has antivirulence activity and effectively inhibits biofilm formation, hemolytic activity, motility, adhesion, invasion and protease activity of the pathogenic bacterium *Aeromonas hydrophila*. The results obtained based on transcriptomic analysis allowed the authors to conclude that EGCG would be a potential alternative drug for the prevention and treatment of *A. hydrophila* infections by antivirulence mechanisms (Yin et al.). Filipić et al. presented a comprehensive overview of currently available *in silico* methods, susceptibility and antivirulence assays, and procedures for testing the cytotoxicity and biosafety of potential antimicrobials. The authors described and assessed methods that have high predictive value and should be used in preclinical studies to identify the most promising antimicrobials (Filipić et al.). In turn, Bear et al. focused on the role of staphylococcal protein A (SpA) which is a central superantigen of *Staphylococcus aureus*. In this review paper the role of protein A in immune evasion was described in terms of mechanisms ranging from dysregulation of the complement cascade to the disruption of leukocyte migration and its negative implications for the persistence of recurrent infections and the development of effective vaccination strategies (Bear et al.). In the context of fast, unambiguous and culture-independent diagnosis of community-acquired methicillin-resistant *S. aureus* (CA-MRSA) infections an important discovery was made by (Yin et al.). Based on label-free quantitative proteomics and non-targeted metabonomics the authors identified potential differentially expressed proteins (DEPs) and metabolites (DEMs) in breast abscesses infected with CA-MRSA compared to methicillin-susceptible *S. aureus* (MSSA). The biomarkers found will certainly allow for the rapid detection of CA-MRSA in breast abscesses in the future (Yin et al.). In conclusion, the papers collected as part of the SI, shed more light on selected virulence factors related to some pathogenic species, potential drugs, antivirulence therapeutic strategies, novel futuristic diagnostics, and the changing epidemiology of some microbial infections.

## Author contributions

MD: Conceptualization, Writing – original draft, Writing – review & editing. BF: Validation, Writing – review & editing. DA: Validation, Writing – review & editing. MM: Validation, Writing – review & editing.
